# Targeting long non-coding RNA PVT1/TGF-β/Smad by p53 prevents glioma progression

**DOI:** 10.1080/15384047.2022.2042160

**Published:** 2022-03-11

**Authors:** Zhang Li, Ming Li, Pengcheng Xia, Lili Wang, Zhiming Lu

**Affiliations:** Department of Clinical Laboratory, Shandong Provincial Hospital, Cheeloo College of Medicine, Shandong University, Jinan,Shandong Province, China

**Keywords:** P53, LncRNA PVT1, TGF-β/Smad pathway, glioma, proliferation, apoptosis

## Abstract

Glioma is a primary intracranial malignant tumor with poor prognosis, and its pathogenesis is unclear. This study discussed the impact of p53/lncRNA plasmacytoma variant translocation 1 (lncRNA PVT1)/transforming growth factor beta (TGF-β)/Smad axis on the biological characteristics of glioma. Glioma and normal tissues were collected, in which relative lncRNA PVT1 and p53 expression was assessed. Pearson’s analysis was adopted for the correlation analysis between lncRNA PVT1 and p53. Short interfering RNA (siRNA) against lncRNA PVT1 (siRNA-PVT1), siRNA-p53 or both was transfected into the glioma cells to evaluate effects of lncRNA PVT1 and p53 on cell proliferation, migration, invasion, and apoptosis. Mouse xenograft model of glioma was established to verify function of lncRNA PVT1 and p53 *in vivo*. Relationship among p53, lncRNA PVT1 and TGF-β/Smad was predicted and confirmed. Glioma tissues and cells showed downregulated p53 expression and increased lncRNA PVT1 expression. An adverse relationship was noted between p53 expression and lncRNA PVT1 expression. p53 was shown to be enriched in the lncRNA PVT1 promoter region and resulted in its suppression. p53 inhibited glioma cell proliferation, migration, and invasion, and induced apoptosis as well as arrested tumor growth by downregulating lncRNA PVT1. LncRNA PVT1was found to bind to TGF-β and activate TGF-β/Smad pathway, promoting progression of glioma. Consequently, p53 exerts anti-oncogenic function on glioma development by suppressing lncRNA PVT1 and subsequently inactivating TGF-β/Smad pathway.

## Introduction

Histologically, there exist over 100 different types of primary brain and central nervous system (CNS) tumors.^1^ Glioma is one most frequent primary brain tumor originating from glial or supporting cells of the CNS, and can be graded I–IV based on morphology and malignant behavior specified in WHO classification.^2^ Glioma can be caused by genetic and environmental factors as well as improper lifestyles, such as inherited mutations, and ionizing radiation.^3^ Current treatment for glioma includes surgery, radiotherapy, chemotherapy, and drug-targeted therapies.^4^ However, there are still high rates of mortality and recurrence, and fatal prognosis.^5^

p53 is considered as a tumor suppressor protein and tightly regulates cell growth *via* promotion of apoptosis and DNA repair under stressful conditions.^6^ Normal function of p53 possesses tumor-suppressing properties, while tumor-associated mutations in p53 are a hallmark of multiple human cancers and lead to significant defects in p53 function.^7^ p53 represents one of the well-documented biomarkers in human glioma owing to its potential of predicting the prognosis in patients with glioma.^8^ Meanwhile, inhibiting p53 expression has been shown to elicit stimulated proliferation of glioma cells, thus aggravating the progression of glioma.^9^ Previous literature has reported lncRNA plasmacytoma variant translocation 1 (lncRNA PVT1) to be a p53-inducible target gene, which produces spliced non-coding RNAs.^10^ Increasing evidence has demonstrated the oncogenic properties of lncRNA PVT1 due to its promoting role in proliferation and growth of many cancers.^11^ LncRNA PVT1 is highly expressed in tumors, including human glioma, where lncRNA PVT1 knockdown attenuates glioma cell viability, migration, and invasion by negatively regulating microRNA-424 (miR-424).^12^ Likewise, miR-128-3p and miR-140-5p have been reported to be implicated in the oncogenic action of lncRNA PVT1.^13–16^ However, the regulatory mechanism underlying high lncRNA PVT1 expression in glioma remains under-studied. Of note, the positive correlation between lncRNA PVT1 and the transforming growth factor beta (TGF-β)/Smad pathway, which acts as a driver of cancer progression by inducing cancer cell viability, metastasis and the epithelial-mesenchymal transition, contributes to pancreatic cancer progression.^17^ Inactivated TGF-β/Smad pathway has been revealed to inhibit glioma cell metastasis,^18^ in addition to advancing glioma cell sensitivity to radiation therapy.^19^ The aforementioned findings suggested a certain correlation among p53, lncRNA PVT1, and TGF-β/Smad pathway, but the knowledge of the mechanism by which they act in glioma is not fully elucidated. Thus, the present study set out for the in-depth analysis of the p53/lncRNA PVT1/TGF-β/Smad pathway in glioma based on *in vitro* and *in vivo* experiments.

## Results

### LncRNA PVT1 was highly expressed in glioma and associated with poor prognosis

**Figure 1.**
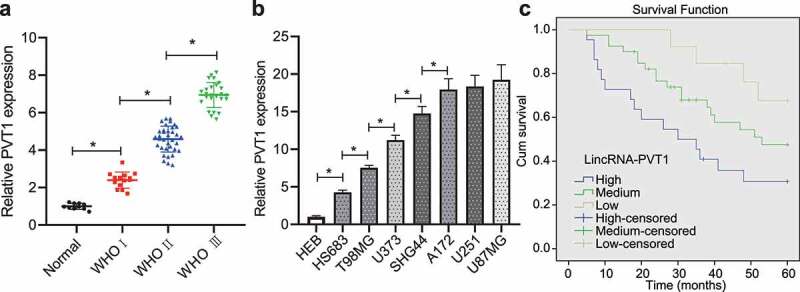
High expression of lncRNA PVT1 in glioma tissues and cells related to poor prognosis. (a), LncRNA PVT1 expression determined by RT-qPCR in clinical samples of patients with glioma (N = 75) and normal samples (N = 10). (b), LncRNA PVT1 expression determined by RT-qPCR in HEB cell line and glioma cell lines. (c), Correlation of lncRNA PVT1 expression with patients’ survival analyzed by the Kaplan-Meier method (N = 75). * *p* < .05. The experiment was repeated 3 times independently.

Initially, lncRNA PVT1 expression was increased in glioma clinical samples from patients at WHO stage I, WHO stage II and WHO stage III while an ascending trend was observed over the staging (Figure 1a). Moreover, upregulation of lncRNA PVT1 was found in HS683, T98, U373, SHG44, A172, U251, and U87MG when compared with HEB cells as determined by RT-qPCR (Figure 1b). These experimental data suggested high lncRNA PVT1 expression in glioma tissues and cells.

Then, patients were divided into three groups according to lncRNA PVT1 expression (low-expression group: lncRNA PVT1 expression was threefold lower, moderate expression group: lncRNA PVT1 expression was three- to sixfold; high-expression group: lncRNA PVT1 expression was sixfold higher than control individuals). Analysis using Kaplan–Meier method showed that patients with higher lncRNA PVT1 expression exhibited shorter survival period (Figure 1c).

### P53 binds to lncRNA PVT1 and inhibits its expression in vitro

**Figure 2.**
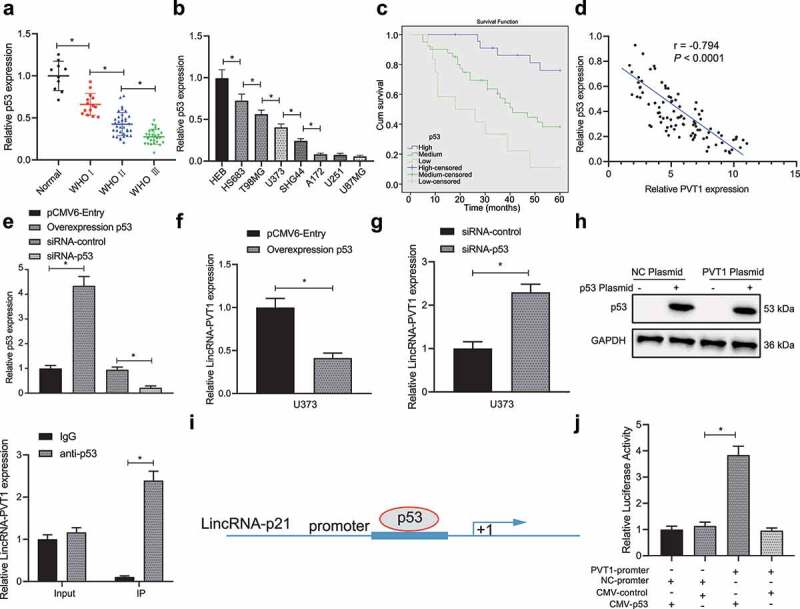
P53 targets lncRNA PVT1. (a), p53 expression determined by RT-qPCR in clinical samples of glioma patients (N = 75) and normal samples (N = 10). (b), p53 expression determined by RT-qPCR in glioma cell lines. (c), Correlation of p53 expression with patients’ survival analyzed by the Kaplan-Meier method (N = 75). (d), Pearson correlation of p53 expression with lncRNA PVT1 expression in clinical samples of patients with glioma (N = 75). (e), Transfection efficiency of oe-p53 and siRNA-p53 determined by RT-qPCR in glioma cells. (f), LncRNA PVT1 expression determined by RT-qPCR in glioma U373 cells following p53 overexpression. (g), LncRNA PVT1 expression determined by RT-qPCR in glioma U373 cells following p53 knockdown. (h), Interaction between lncRNA PVT1 and p53 confirmed by RIP assay in glioma cells. (i), Binding sites between lncRNA PVT1 promoter and p53 predicted on lncATLAS website. (j), Binding of p53 to lncRNA PVT1 promoter confirmed by dual-luciferase reporter gene assay in 293 T cells. * *p* < .05. The experiment was repeated 3 times independently.

Subsequently, p53 expression in clinical glioma samples was determined. As shown in Figure 2a, p53 was downregulated in 3 WHO stages and the decline was more significant over the tumor progression. In addition, low expression of p53 was detected in HS683, T98, U373, SHG44, A172, U251, and U87MG cell lines when compared with HEB cell line as revealed by RT-qPCR (Figure 2b).

Then, patients were divided into three groups according to p53 expression (high-expression group: p53 expression was 1/2 higher, moderate expression group: p53 expression was 1/2 – 1/4-fold; low-expression group: p53 expression was 1/4-fold lower than control individuals). Kaplan–Meier analysis showed that higher p53 expression corresponded to the longer survival period of patients (Figure 2c). Further Pearson’s correlation coefficient revealed an inverse relation between lncRNA PVT1 and p53 expression in glioma (Figure 2d).

In order to further examine the relationship between lncRNA PVT1 and p53, we transfected glioma U373 cell line (moderate lncRNA PVT1 and p53 expression) for silence/overexpression of p53, and then examined their transfection efficiency by RT-qPCR (Figure 2e). In addition, decreased lncRNA PVT1 expression was observed in U373 cells following p53 overexpression, while p53 knockdown elicited upregulated lncRNA PVT1 expression (Figure 2f, g).

Furthermore, the RIP assay revealed an interaction between lncRNA PVT1 and p53 in U373 cells (Figure 2h). In order to investigate on whether p53 regulated lncRNA PVT1 transcription, lncATLAS website (http://lncatlas.crg.eu/) predicted a putative binding region of p53 in lncRNA PVT1 promoter (Figure 2i). Dual-luciferase reporter gene assay provided further evidence that luciferase activity was increased by lncRNA PVT1 promoter and CMY-p53 plasmids (Figure 2j).

### P53 impedes glioma cell proliferation, migration, and invasion, while inducing apoptosis by targeting lncRNA PVT1

**Figure 3.**
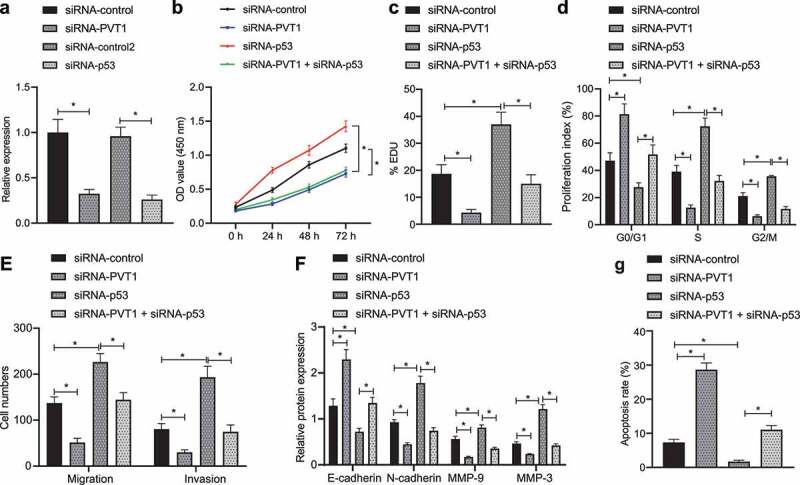
P53 targeting lncRNA PVT1 restrains glioma cell proliferation, migration, and invasion, whereas inducing apoptosis *in vitro*. (a), Transfection efficiency of siRNA-PVT1 and siRNA-p53 determined by RT-qPCR in U373 cells. (b), U373 cell viability measured by CCK-8 assay. (c), U373 cell proliferation measured by EdU assay. (d), U373 cell cycle distribution detected by flow cytometry. (e), U373 cell migration and invasion measured by Transwell assay. (f), Expression of N-cadherin, MMP3, E-cadherin, and MMP9 proteins in U373 cells determined by Western blot analysis. (g), U373 cell apoptosis measured by flow cytometry. * *p* < .05. The experiment was repeated 3 times independently.

Next, we aimed to investigate whether p53 can inhibit the biological behavior of glioma cells by targeting lncRNA PVT1. We transfected the representative glioma U373 cell lines with siRNA-PVT1 or siRNA-p53 for 48 h and then verified their transfection efficiency by conducting RT-qPCR (Figure 3a).

According to CCK-8 assay (Figure 3b), EdU assay (Figure 3c), and flow cytometry (Figure 3d), transfection with siRNA-PVT1 decreased cell viability and proliferation, arrested cells at S and G_2_/M phases, and reduced cell arrest at G0/G1 phase, all of which were found to be opposite following transfection of siRNA-p53 alone, while treatment of both siRNA-PVT1 and siRNA-p53 abolished the action of siRNA-p53 alone. Taken together, p53 inhibited glioma cell proliferation by suppressing lncRNA PVT1 *in vitro*.

Figure 3e illustrates a descending cell migration and invasion trend following transfection with siRNA-PVT1, while transfection with siRNA-p53 brought about a contrasting trend, and treatment of both siRNA-PVT1 and siRNA-p53 abolished the action of siRNA-p53 alone. Meanwhile, Western blot analysis (Figure 3f) showed that N-cadherin, MMP3, and MMP9 expression was reduced, while E-cadherin expression was elevated in siRNA-PVT1-transfected cells, while transfection with siRNA-p53 induced opposite trends, and additional transfection of siRNA-PVT1 counterweighed the effect of siRNA-p53 (Figure 3e, F; Supplementary Figure 1). These results suggested that glioma cell migration and invasion were repressed by p53-mediated lncRNA PVT1 inhibition *in vitro*.

Subsequently, we attempted to explore whether p53 can mediate glioma cell apoptosis by targeting lncRNA PVT1 using flow cytometry. It was found that cell apoptosis was suppressed by siRNA-p53 yet promoted by siRNA-PVT1, while co-transfection of siRNA-p53 and siRNA-PVT1 neutralized the function of siRNA-p53 (Figure 3g), highly suggestive of the promoting action of p53 on glioma cell apoptosis by targeting lncRNA PVT1.

### P53 inactivates the TGF-β/Smad pathway in glioma cells by targeting lncRNA PVT1

**Figure 4.**
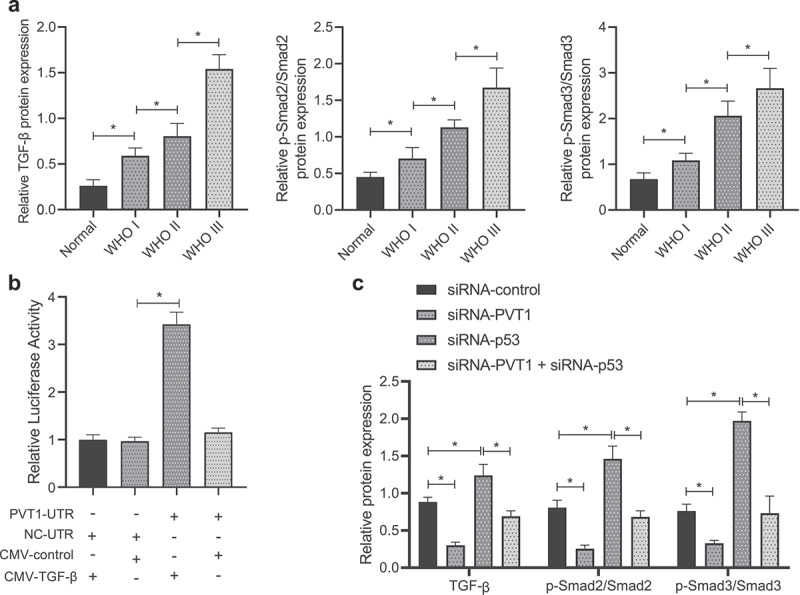
P53 mediates the TGF-β/Smad pathway by targeting lncRNA PVT1. (a), Expression of the TGF-β/Smad pathway-related proteins in clinical samples of patients with glioma (N = 75) and normal samples (N = 10) determined by Western blot analysis. (b), Binding between TGF-β and lncRNA PVT1 analyzed by dual-luciferase reporter gene assay. (c), Expression of the TGF-β/Smad signaling pathway-related proteins in U373 cells determined by Western blot analysis. * *p* < .05. The experiment was repeated 3 times independently.

Then, TGF-β/Smad pathway-related protein expression was measured in clinical glioma samples (Figure 4a; Supplementary Figure 2A), results of which revealed upregulation of TGF-β and increased extent of Smad2/3 phosphorylation in glioma samples, while the increase tended to be more significant over the glioma stages.

Further investigation revealed that the luciferase activity was increased in cells co-transfected with lncRNA PVT1 UTR and CMY-TGF-β plasmids (Figure 4b). Moreover, TGF-β expression and extent of Smad2/3 phosphorylation were attenuated in glioma cells transfected with siRNA-PVT1, while siRNA-p53 transfection led to opposite effects, and co-treatment of siRNA-p53 and siRNA-PVT1 counteracted the effect of siRNA-p53 (Figure 4c).

### P53 arrests tumor growth of glioma via inhibition on the lncRNA PVT1/TGF-β/Smad axis

**Figure 5.**
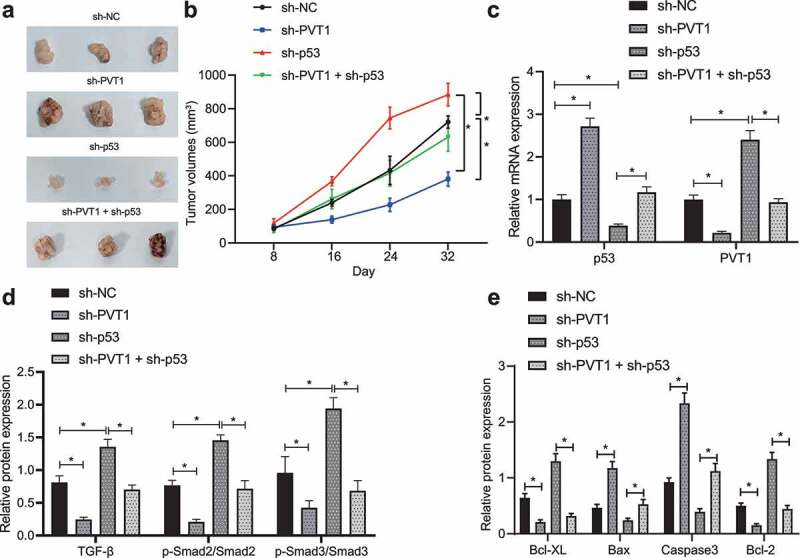
P53 targeting lncRNA PVT1 prevents tumor growth and curbs apoptosis *in vivo* through the TGF-β/Smad pathway. (a), Representative images showing xenografts in nude mice injected with U373 cells transfected with sh-PVT1, sh-p53 or both. (b), Growth of human glioma xenogratf tumor measured every 8 days in nude mice injected with U373 cells transfected with sh-PVT1, sh-p53 or both. (c), Expression of p53 and lncRNA PVT1 in tumor tissues determined by Western blot analysis. (d), Expression of TGF-β and Smad2/3 and the extent of Smad2/3 phosphorylation in tumor tissues determined by Western blot analysis. (e), Expression of Caspase-3, Bax, Bcl-xL, and Bcl-2 proteins determined by Western blot analysis. N = 6 for mice upon each treatment. * *p* < .05. The experiment was repeated 3 times independently.

Finally, we established a glioma model in mice using U373 cells transfected with sh-p53, sh-PVT1 or both to evaluate the effect of p53 target-inhibition of lncRNA PVT1 on glioma. By measuring tumor growth, tumor growth was inhibited in mice treated with sh-PVT1 alone but sh-p53 alone increased tumor growth, and combined treatment with sh-p53 + sh-PVT1 treatment abolished the action of sh-p53 alone (Figure 5a, b).

After 32 days, nude mice were euthanized to retrieve tumors for subsequent analysis. Expression of p53, lncRNA PVT1, TGF-β and extent of Smad2/3 phosphorylation was determined in tumor tissues (Figure 5c, d). In response to sh-p53 treatment, p53 expression was reduced, while the expression of lncRNA PVT1, TGF-β and extent of Smad2/3 phosphorylation was elevated. Opposite results were observed in presence of sh-PVT1. Also, action of sh-p53 was counterweighed by further sh-PVT1 treatment.

The pro-apoptotic Caspase-3 and Bax expression was upregulated, but that of anti-apoptotic Bcl-xL and Bcl-2 genes was decreased in mouse tumor tissues upon transfection with sh-PVT1, while sh-p53 treatment induced opposite changing tendency, and combined treatment with sh-p53 + sh-PVT1 counteracted and action of sh-p53 treatment alone (Figure 5e; Supplementary Figure 2B).

## Discussion

p53 is one of the key anti-oncogenes in multiple cancers, the activity of which is ubiquitously lost in human cancers by p53 gene mutation (60% of cancers) or by the loss of cell signaling upstream and downstream of p53 in the remaining cancers expressing p53-wild type gene.^20^ In the current study, we demonstrated that p53 potentially contributed to lncRNA PVT1-mediated TGF-β/Smad pathway inactivation and therefore inhibiting the occurrence and progression of glioma, revealing that p57 and lncRNA PVT1 were functionally correlated through a binding relation in glioma.

p53 expression has been verified to be remarkably decreased in tumor tissue specimens of patients with glioma relative to adjacent normal tissue specimens.^21^ The present study also found downregulated p53 expression in glioma tissues and cells. p53, an essential tumor suppressor, prevents tumor progression; a recent study has indicated that inhibition of p53 expression induced by LINC00467 upregulation promotes proliferation and invasion abilities of glioma cells.^22^ In addition, activated p53 confers an inhibitory effect on glioma tumor growth following a xenograft tumor model construction.^23^ These findings suggested that p53 may be a potential target for diagnostics and therapeutics in glioma. Similar to our results, p53 possesses the characteristics to bind to the lncRNA PVT1 promoter and divergently regulates transcription efficiency of lncRNA PVT1 in hepatocellular carcinoma cells; specifically, upregulated p53 decreased lncRNA PVT1 expression in SMMC-7721 and HepG2 cells.^24^ LncRNA PVT1 acts as an oncogenic regulator of multiple malignancies, highly expressed in many malignancies and suggests a poor prognosis, including glioma.^25^ For instance, lncRNA PVT1 is abundantly expressed in glioma clinical samples, and has superior diagnostic values as the glioma patients with high lncRNA PVT1 expression exhibit worse prognosis.^15^ lncRNA PVT1 expression is observed to be much higher in glioma tissues, and higher in high grade (III–IV) than low grade (I–II) tumors.^26^ Also, lncRNA PVT1 is a promising biomarker for diagnosis, therapy, and prognosis of patients with diffuse glioma.^27^ Meanwhile, attenuated lncRNA PVT1 suppressed proliferation, migration and invasion, and boosted apoptosis of U251 and U87 cells along with reduced transplanted tumor volume and weight of glioma in mice.^14,28^ The aforementioned findings concur with our findings that p53 had the potential to impede glioma cell proliferation, migration, and invasion while inducing apoptosis and arresting tumor growth *via* lncRNA PVT1 inhibition.

Overexpression of PVT1 has been found to activate TGF-β/Smad pathway in pancreatic cancer cells.^17^ In this study, we identified that lncRNA PVT1 activated TGF-β/Smad pathway in glioma cells. TGF-β triggers tumor pathogenesis possibly by directly supporting tumor growth, maintaining self-renewal of glioma initiating stem cells or inhibiting anti-tumor immunity.^29^ Activation of the TGF-β/Smad pathway predicts a poor prognosis since patients with activated TGF-β/Smad show a short overall survival.^30^ Poly (C)-binding protein 2 activates TGF-β/Smad pathway *via* inhibition of familial hemophagocytic lymphohistiocytosis 3, and thus aggravates glioma.^31^ Moreover, repressing TGF-β/Smad pathway helps to inhibit proliferation and promote apoptosis of glioma cells, thus ameliorating glioma and other related diseases.^32^ p53 can inhibit TGF-β expression and induce the resultant suppressed migration and growth of human Tenon’s fibroblasts.^33^ Downregulation of the TGF-β1/Smad/p38 pathway activation contributes to increased p53 expression and subsequently suppresses proliferation of pulmonary arterial smooth muscle cells, ultimately leading to retarded pulmonary arterial hypertension.^34^ Based on the findings above, we reasoned that p53 silencing might promote lncRNA PVT1-mediated TGF-β/Smad pathway activation and accelerate glioma.

**Figure 6.**
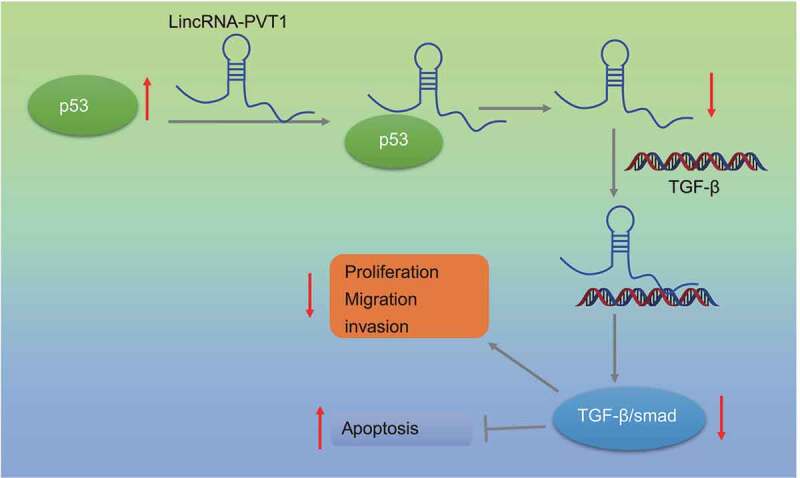
Mechanism diagram displaying the effect of the p53-mediated lncRNA PVT1/TGF-β/Smad axis on the proliferation, migration, invasion, and apoptosis of glioma cells.

In summary, we identified a novel regulatory signaling axis, p53/lncRNA PVT1/TGF-β/Smad, in glioma (Figure 6). Our findings may lead to rational drug discovery specifically targeting this axis in human glioma. Nevertheless, due to some reports on the positive correlation between p53 and the TGF-β/Smad pathway,^35,36^ additional studies are required to investigate specific mechanism of this axis in glioma.

## Methods

### Ethics statement

The study was approved by Ethics Committee of Shandong Provincial Hospital, Cheeloo College of Medicine, Shandong University. All patients have received written informed consents prior to sample collection. Animal experiments were conducted under approval of the Ethics Committee of Shandong Provincial Hospital, Cheeloo College of Medicine, Shandong University.

### Sample collection

Primary tumor tissue samples were collected from 75 patients with glioma underwent surgery at the Department of Neurosurgery of Shandong Provincial Hospital, Cheeloo College of Medicine, Shandong University from January 2010 to August 2014, followed by pathological examination. Based on the 2000 WHO classification of glioma, there were 14 cases at stage I, 35 cases at stage II, and 26 cases at stage III. Ten normal brain tissue samples were obtained from patients received intracranial decompression surgery with brain swelling and brain hernia formation after craniocerebral trauma, serving as the control. After collection, all specimens were immediately frozen in liquid nitrogen and then stored at −80°C.

### RT-qPCR

Total RNA was extracted using TRIzol reagents (15596026, Invitrogen, Carlsbad, CA, USA). The extracted RNA was reversely transcribed into cDNA using the PrimeScript RT reagent Kit (RR047A, Takara, Japan). Next, synthesized cDNA was subjected to RT-qPCR using the Fast SYBR Green PCR kit (Applied Biosystems, Foster City, CA, USA) on an ABI PRISM 7300 instrument (Applied Biosystems). The primers are all listed in Supplementary Table 1. The fold changes of target genes were calculated by 2^−ΔΔCt^ method.

### Cell treatment

Human renal epithelial cells 293 T (257191), normal brain tissue cells HEB (MZ-0831), and glioma cell lines U373 (08061901, ECACC General Collection), U87MG (89081402, ECACC General Collection), HS683 (MZ-1343, Mingzhou Biotech Co., Ltd., Ningbo, Zhejiang, China), T98 (MZ-0789, Mingzhou Biotech), A172 (MZ-0012, Mingzhou Biotech), SHG-44, and U251MG (MZ-2062, Mingzhou Biotech) were cultured in RPMI 1640 medium (Gibco Company, NY, USA) at 37°C with 5% CO_2_.

The cells were trypsinized, inoculated into 6-well plates at 1 × 10^5^ cells/well, and cultured for 24 h. Upon 75% confluence, cells were transfected using Lipofectamine 3000 reagents (Invitrogen). To overexpress lncRNA PVT1, lncRNA PVT1 cDNA was amplified by RT-qPCR and sub-cloned into pcDNA™3.1 vector (Thermo Fisher Scientific). The sequence of small interfering RNA (siRNA) targeting lncRNA PVT1 (siRNA-PVT1; sense: 5’-UUAGUAUCCUGAAAUGUGC-3’, antisense: 5’-GCACAUUUCAGGAUACUAA-3’) and the negative control (NC) sequence (siRNA-control; sense: 5’-UUCUCCGAACGUGUCACGUTT-3’, antisense: 5’-ACGUGACACGUUCGGAGAATT-3’) were designed.^28^ The sequence of siRNA targeting p53 (SR322075), consisting of siRNA-control (sense: 5’-ACUACUGAGUGACAGUAGA-3’, antisense: 5’-UCUACUGUCACUCAGUAGU-3’) and siRNA-p53 (sense: 5’-UGCGUGUGGAGUAUUUGGAUG-3’, antisense: 5’-UGGUACAGUCAGAGCCAACCUC-3’), was designed by OriGene Company. The corresponding treatment was conducted 48 h after transfection.

### Dual luciferase reporter gene assay

First, upstream 2000 base sequences of lncRNA PVT1 transcription starting point was cloned into pGL4.0-Basic luciferase vector (Obio Technology, Shanghai, China) to construct fluorescent plasmids with reference to the manual of dual luciferase reporter system (Promega, Madison, WI, USA). The plasmids were then co-transfected into 293 T cells with p53 overexpression plasmid (CMV-p53).

The 3ʹuntranslated region of mouse TGF-β containing the putative lncRNA PVT1 binding sites was amplified from mouse genomic DNA by RT-qPCR, and then inserted into pmiR-REPORT (Guangzhou RiboBio, Guangdong, China) to construct fluorescent plasmids, which were co-transfected with TGF-β overexpression plasmids (CMV-TGF-β) into 293 T cells.

After 48 h of transfection, luciferase activity was measured using the Dual Luciferase Reporter Assay System (Promega).

### RNA-Binding protein immunoprecipitation (RIP) assay

The binding of lncRNA PVT1 to p53 protein was detected by the RIP Assay Kit (Millipore, Billerica, MA, USA). U373 cells were lysed with radioimmunoprecipitation assay (RIPA) lysis solution (P0013b, Beyotime, Shanghai, China) for 5 min, and then centrifuged at 14000 rpm and 4°C for 10 min. The supernatant was collected and divided into two equal parts, one of which was taken out as input, and the other incubated with rabbit antibodies against p53 (#2527, Cell Signaling Technologies, Beverly, MA, USA) for 30 min, and rabbit anti-human immunoglobulin G (IgG; 1: 100, ab109489, Abcam, Cambridge, UK) serving as NC for co-precipitation. The sample and input were detached by protease K, and RNA was then extracted for RT-qPCR detection of lncRNA PVT1 expression.

### CCK-8 assay

The cells were collected and inoculated into 96-well plates at 3 × 10^3^ cells/well. After adhered to the wall, CCK-8 reaction solution (341–07761, Dojindo Laboratories, Kumamoto, Japan) was added at 24, 48, and 72 h, and then incubated for 2 h. The optical value value was measured at 450 nm using a microplate reader. Seven parallel wells were set.

### EdU assay

The cells were plated into 24-well plates. The culture medium was added with EdU solution (C10310-1, RiboBio) to a concentration of 10 µmol/L, and cells were then incubated for 2 h. Next, cells were fixed, washed with PBS containing 3% bovine serum albumin (BSA), and incubated with PBS containing 0.5% Triton-100 for 20 min. Each well was added with 100 μL dye solution and incubated for 30 min. Following nuclear staining with DAPI for 5 min, the slide was mounted and number of positive cells was counted under a fluorescence microscope (FM-600, Pudan Optical Instrument, Shanghai, China).

### Flow cytometry

After treatment, cells were detached with trypsin and collected. Next, cells were fixed overnight with 75% alcohol. The next day, approximately 100 μL cell suspension containing 10^5^ cells was collected and treated according to protocols on the Muse™ Cell Cycle Kit or Muse® Annexin V & Dead Cell Kit, followed by analysis on a flow cytometer (MUSE MILLIPORE) to analyze proportion of cells arrested at G0/G1, S and G2/M phases as well as proportion of apoptotic cells.

### Transwell assay

The upper Transwell chamber was coated with Matrigel (BD Biosciences, Franklin Lakes, NJ, USA). The cells were cultured in serum-free medium for 12 h and resuspended in serum-free medium at 1 × 10^5^ cells/mL. Then the medium containing 10% FBS was put into lower chamber, which was added with 100 μL cell suspension for incubation at 37°C for 24 h. The cells that did not invade surface of Matrigel membrane were removed, the rest was fixed with 100% methanol and stained with 1% toluidine blue (Sigma-Aldrich, St Louis, MO, USA). The stained invasive cells were counted under an inverted light microscope (Carl Zeiss, Oberkochen, Germany). Cell migration was detected without Matrigel coating.

### Western blot analysis

Total protein was isolated using RIPA lysis buffer, separated by 10% sodium dodecyl sulfate polyacrylamide gel electrophoresis, and then transferred onto polyvinylidene fluoride membranes. The membrane was and probed with diluted primary rabbit antibodies against Caspase-3 (ab13847), Bcl-2-associated X protein (Bax; ab32503), B-cell lymphoma 2 (Bcl-2) (ab32124), Bcl-XL (ab32370), TGF-β1 (ab92486), β-actin (ab8227), GAPDH (ab181602), E-cadherin (3195), N-cadherin (13116), matrix metalloproteinase (MMP)3 (14351), MMP9 (13667), Smad2 (5339), Smad3 (9523), phosphorylated (p)-Smad2 (18338), and p-Smad3 (9520, Cell Signaling Technologies) overnight at 4°C. The antibodies were from Abcam except for p-Smad2/3. After washing, membrane was re-probed with the horseradish peroxidase-conjugated goat anti-rabbit secondary antibody (ab205719, 1: 2000, Abcam) for 1 h. The protein bands were visualized using enhanced chemiluminescence (EMD Millipore). β-actin and GAPDH were used as internal controls. The gray values were analyzed with Image J software.

### Nude mouse models of glioma

A total of 24 female 4-week BALB/c nude mice purchased from Beijing Institute of Pharmacology and Toxicology, Chinese Academy of Medical Sciences were selected in this study, and individually housed in specific pathogen-free animal laboratory at 22–25°C, humidity of 60% – 65%, and a 12-h light/12-h dark cycle with free access to food and water. After one week of adaptive feeding, mice were subjected to subsequent experiments.

Lentivirus harboring short hairpin RNA (shRNA) against lncRNA PVT1 (sh-PVT1), sh-p53 or sh-NC (Sigma-Aldrich) was titrated to 10^9^ TU/mL. U373 cells were seeded in a 6-well plate at 2 × 10^5^ cells/well and cultured for 24 h. U373 cells were infected with lentivirus for 72 h and cultured at least 14 days in medium containing 4 μg/mL puromycin. Cells resistant to puromycin were cultured for 9 days in medium containing 2 μg/mL puromycin and further cultured in puromycin-free medium to collect U373 cells with stable knockdown of lncRNA PVT1 or p53.

Nude mice were injected with 2 × 10^6^ U373 cells that had been transfected with plasmids of shRNA-p53, shRNA-PVT1, or both at the armpit of upper limb. The diameter of tumor was measured twice every 8 days. Thirty two days later, mice were euthanized, after which tumor volume and weight were assessed.

### Statistical analysis

All statistical analyses were conducted using SPSS 21.0 (IBM, NY, USA). The measurement data were presented as mean ± standard deviation. Data between two groups were compared using unpaired *t*-test and those among multiple groups were compared using one-way ANOVA, followed by Tukey multiple comparisons posttest. Cell viability at different time points was compared by two-way ANOVA, tumor data at different time points were analyzed by Bonferroni-corrected repeated measures ANOVA. The survival rate was calculated by the Kaplan-Meier method and analyzed by Log-rank test. Pearson’s correlation coefficient was used to analyze correlation of two indexes. *p* < .05 was considered statistically significant.

## Supplementary Material

Supplemental Material

## References

[1] Davis ME. 2018. Epidemiology and overview of gliomas. Semin Oncol Nurs. 34(5):420–429. doi:10.1016/j.soncn.2018.10.001. 30392758

[2] Rasmussen BK, Hansen S, Laursen RJ, Kosteljanetz M, Schultz H, Norgard BM, Guldberg R, Gradel KO. 2017. Epidemiology of glioma: clinical characteristics, symptoms, and predictors of glioma patients grade I-IV in the the danish neuro-oncology registry. J Neurooncol. 135(3):571–579. doi:10.1007/s11060-017-2607-5. 28861666

[3] Bielecka J, Markiewicz-Zukowska R. 2020. The influence of nutritional and lifestyle factors on glioma incidence. Nutrients. 12(6):1812. doi:10.3390/nu12061812. PMC7353193 32560519

[4] Miller JJ, Wen PY. 2016. Emerging targeted therapies for glioma. Expert Opin Emerg Drugs. 21(4):441–452. doi:10.1080/14728214.2016.1257609. 27809598

[5] Wang X, Jia Y, Wang P, Liu Q, Zheng H. 2017. Current status and future perspectives of sonodynamic therapy in glioma treatment. Ultrason Sonochem. 37:592–599. doi:10.1016/j.ultsonch.2017.02.020. 28427672

[6] Kanapathipillai M. 2018. Treating p53 mutant aggregation-associated cancer. Cancers (Basel). 10(6):154. doi:10.3390/cancers10060154. PMC6025594 29789497

[7] Whibley C, Pharoah PD, Hollstein M. 2009. p53 polymorphisms: cancer implications. Nat Rev Cancer. 9(2):95–107. doi:10.1038/nrc2584. 19165225

[8] Jin Y, Xiao W, Song T, Feng G, Expression DZ. 2016. Prognostic significance of p53 in glioma patients: a meta-analysis. Neurochem Res. 41(7):1723–1731. doi:10.1007/s11064-016-1888-y. 27038932

[9] Zhao QS, Ying JB, Jing JJ, Wang SS. 2020. LncRNA FOXD2-AS1 stimulates glioma progression through inhibiting P53. Eur Rev Med Pharmacol Sci. 24(8):4382–4388. doi:10.26355/eurrev_202004_21019. 32373975

[10] Barsotti AM, Beckerman R, Laptenko O, Huppi K, Caplen NJ, Prives C. 2012. p53-Dependent induction of PVT1 and miR-1204. J Biol Chem. 287(4):2509–2519. doi:10.1074/jbc.M111.322875. 22110125 PMC3268411

[11] Derderian C, Orunmuyi AT, Olapade-Olaopa EO, Ogunwobi OO. 2019. PVT1 signaling is a mediator of cancer progression. Front Oncol. 9:502. doi:10.3389/fonc.2019.00502. 31249809 PMC6582247

[12] Han Y, Li X, He F, Yan J, Ma C, Zheng X, Zhang J, Zhang D, Meng C, Zhang Z, et al. 2019. Knockdown of lncRNA PVT1 inhibits glioma progression by regulating miR-424 expression. Oncol Res. 27(6):681–690. doi:10.3727/096504018X15424939990246. 30832754 PMC7848267

[13] Fu C, Li D, Zhang X, Liu N, Chi G, Jin X. 2018. LncRNA PVT1 facilitates tumorigenesis and progression of glioma via regulation of MiR-128-3p/GREM1 Axis and BMP signaling pathway. Neurotherapeutics. 15(4):1139–1157. doi:10.1007/s13311-018-0649-9. 30120709 PMC6277294

[14] Dahai Z, Daliang C, Famu L, Xiang W, Lenian L, Jianmin C, Xiaobing X. 2020. Lowly expressed lncRNA PVT1 suppresses proliferation and advances apoptosis of glioma cells through up-regulating microRNA-128-1-5p and inhibiting PTBP1. Brain Res Bull. 163:1–13. doi:10.1016/j.brainresbull.2020.06.006. 32562719

[15] Shao Y, Chen HT, Ma QR, Zhang YW, He YQ, Liu J. 2020. Long non-coding RNA PVT1 regulates glioma proliferation, invasion, and aerobic glycolysis via miR-140-5p. Eur Rev Med Pharmacol Sci. 24(1):274–283. doi:10.26355/eurrev_202001_19922. 31957841

[16] Shao Y, Chen HT, Ma QR, Zhang YW, He YQ, Liu J. 2020. Long non-coding RNA PVT1 regulates glioma proliferation, invasion, and aerobic glycolysis via miR-140-5p. Eur Rev Med Pharmacol Sci. 24(16):8249. doi:10.26355/eurrev_202008_22590. 32894529

[17] Zhang X, Feng W, Zhang J, Ge L, Zhang Y, Jiang X, Peng W, Wang D, Gong A, Xu M. 2018. Long noncoding RNA PVT1 promotes epithelialmesenchymal transition via the TGFbeta/Smad pathway in pancreatic cancer cells. Oncol Rep. 40(2):1093–1102. doi:10.3892/or.2018.6462. 29845201

[18] Guan F, Kang Z, Wang L, Wang K, Mao BB, Peng WC, Zhang BL, Lin ZY, Zhang JT, Hu ZQ. 2019. Retinol dehydrogenase 10 promotes metastasis of glioma cells via the transforming growth factor-beta/SMAD signaling pathway. Chin Med J (Engl). 132(20):2430–2437. doi:10.1097/CM9.0000000000000478. 31613821 PMC6831065

[19] Tao S, Liu M, Shen D, Zhang W, Wang T, Bai Y. 2018. TGF-beta/Smads signaling affects radiation response and prolongs survival by regulating DNA repair genes in malignant glioma. DNA Cell Biol. 37(11):909–916. doi:10.1089/dna.2018.4310. 30230914

[20] Bourdon JC. 2007. p53 and its isoforms in cancer. Br J Cancer. 97(3):277–282. doi:10.1038/sj.bjc.6603886. 17637683 PMC2360320

[21] Yu BX, Zou L, Li S, Du YL. 2019. LncRNA SAMD12-AS1 down-regulates P53 to promote malignant progression of glioma. Eur Rev Med Pharmacol Sci. 23(19):8456–8467. doi:10.26355/eurrev_201910_19158. 31646576

[22] Zhang Y, Jiang X, Wu Z, Hu D, Jia J, Guo J, Tang T, Yao J, Liu H, Tang H. 2020. Long Noncoding RNA LINC00467 promotes glioma progression through inhibiting P53 expression via binding to DNMT1. J Cancer. 11(10):2935–2944. doi:10.7150/jca.41942. 32226508 PMC7086258

[23] Zhen ZG, Ren SH, Ji HM, Ma JH, Ding XM, Feng FQ, Chen SL, Zou P, Ren JR, Jia L. 2017. Linarin suppresses glioma through inhibition of NF-kappaB/p65 and up-regulating p53 expression in vitro and in vivo. Biomed Pharmacother. 95:363–374. doi:10.1016/j.biopha.2017.08.023. 28858735

[24] Xiong X, Yuan J, Zhang N, Zheng Y, Liu J, Yang M. 2020. Silencing of lncRNA PVT1 by miR-214 inhibits the oncogenic GDF15 signaling and suppresses hepatocarcinogenesis. Biochem Biophys Res Commun. 521(2):478–484. doi:10.1016/j.bbrc.2019.10.137. 31677796

[25] Pan X, Zheng G, Gao C. 2018. LncRNA PVT1: a novel therapeutic target for cancers. Clin Lab. 64(5):655–662. doi:10.7754/Clin.Lab.2018.171216. 29739059

[26] Fang J, Huang J. 2019. Clinical significance of the expression of long non-coding RNA PVT1 in glioma. Cancer Biomark. 24(4):509–513. doi:10.3233/CBM-182253. 30909189 PMC13082533

[27] Zou H, Wu LX, Yang Y, Li S, Mei Y, Liu YB, Zhang L, Cheng Y, Zhou HH. 2017. lncRNAs PVT1 and HAR1A are prognosis biomarkers and indicate therapy outcome for diffuse glioma patients. Oncotarget. 8(45):78767–78780. doi:10.18632/oncotarget.20226. 29108264 PMC5667997

[28] Yang A, Wang H, Yang X. Long non-coding RNA PVT1 indicates a poor prognosis of glioma and promotes cell proliferation and invasion via target EZH2. Biosci Rep. 2017;37(6). doi:10.1042/BSR20170871. PMC6435466 29046366

[29] Kaminska B, Cyranowski S. 2020. Recent advances in understanding mechanisms of TGF beta signaling and its role in glioma pathogenesis. Adv Exp Med Biol. 1202:179–201. doi:10.1007/978-3-030-30651-9_9. 32034714

[30] Sferra R, Pompili S, Festuccia C, Marampon F, Gravina GL, Ventura L, Di Cesare E, Cicchinelli S, Gaudio E, Vetuschi A. 2017. The possible prognostic role of histone deacetylase and transforming growth factor beta/Smad signaling in high grade gliomas treated by radio-chemotherapy: a preliminary immunohistochemical study. Eur J Histochem. 61(2):2732. doi:10.4081/ejh.2017.2732. 28735518 PMC5439439

[31] Mao J, Sun Z, Cui Y, Du N, Guo H, Wei J, Hao Z, Zheng L. 2020. PCBP2 promotes the development of glioma by regulating FHL3/TGF-beta/Smad signaling pathway. J Cell Physiol. 235(4):3280–3291. doi:10.1002/jcp.29104. 31693182 PMC7166520

[32] Jin H, Luo C. Bleomycin inhibits proliferation and promotes apoptosis of brain glioma cells via TGF-beta/Smad signaling pathway. J BUON. 2020;25(2):1076–1083. 32521909

[33] Li N, Zhang R. 2020. Silencing of p53 reduces cell migration in human Tenon’s fibroblasts induced by TGF-beta. Int Ophthalmol. 40(6):1509–1516. doi:10.1007/s10792-020-01320-9. 32124130

[34] Yue Y, Li YQ, Fu S, Wu YT, Zhu L, Hua L, Lv JY, Li YL, Yang DL. 2020. Osthole inhibits cell proliferation by regulating the TGF-beta1/Smad/p38 signaling pathways in pulmonary arterial smooth muscle cells. Biomed Pharmacother. 121:109640. doi:10.1016/j.biopha.2019.109640. 31810114

[35] Wiener Z, Band AM, Kallio P, Hogstrom J, Hyvonen V, Kaijalainen S, Ritvos O, Haglund C, Kruuna O, Robine S, et al. 2014. Oncogenic mutations in intestinal adenomas regulate Bim-mediated apoptosis induced by TGF-beta. Proc Natl Acad Sci U S A. 111(21):E2229–2236. doi:10.1073/pnas.1406444111. 24825889 PMC4040601

[36] Al-Shabanah OA, Aleisa AM, Hafez MM, Al-Rejaie SS, Al-Yahya AA, Bakheet SA, Al-Harbi MM, Sayed-Ahmed MM. 2012. Desferrioxamine attenuates doxorubicin-induced acute cardiotoxicity through TFG-beta/Smad p53 pathway in rat model. Oxid Med Cell Longev. 2012:619185. doi:10.1155/2012/619185. 22619697 PMC3350848

