# Supplementary material for: Targeting long non-coding RNA PVT1/TGF-β/Smad by p53 prevents glioma progression
Source: Cancer Biol Ther. 2022 Mar 11;23(1):225–33. doi: 10.1080/15384047.2022.2042160 (PMC8920172; doi:10.1080/15384047.2022.2042160)
Supplement: Supplemental Material [file KCBT_A_2042160_SM8502.zip › supplementary/Supplementary Table 1.docx]

**Supplementary Table 1.** Primer sequences for RT-qPCR

| Gene | Sequence |
| --- | --- |
| p53 | F: 5'-GCTCGACGCTAGGATCTGAC-3' |
|  | R: 5'-CAGGTAGCTGCTGGGCTC-3' |
| lncRNA PVT1 | F: 5'-GCCCTCCAGCCTGATCTTTT-3' |
|  | R: 5'-TTCCACCAGCGTTATTCCCC-3' |
| GAPDH | F: 5'-GACTCATGACCACAGTCCATGC-3' |
|  | R: 5'-AGAGGCAGGGATGATGTTCTG-3' |

Note: RT-qPCR, reverse transcription quantitative polymerase chain reaction; F, forward; R, reverse; lncRNA PVT1, long non-coding RNA plasmacytoma variant translocation 1; GAPDH, glyceraldehyde-3-phosphate dehydrogenase.
